# GZ17-6.02 and axitinib interact to kill renal carcinoma cells

**DOI:** 10.18632/oncotarget.28189

**Published:** 2022-02-04

**Authors:** Laurence Booth, Cameron West, Robert P. Moore, Daniel Von Hoff, Paul Dent

**Affiliations:** ^1^Department of Biochemistry and Molecular Biology, Virginia Commonwealth University, Richmond, VA 23298, USA; ^2^Genzada Pharmaceuticals, Sterling, KS 67579, USA; ^3^Physician-in-Chief, Distinguished Professor, Translational Genomics Research Institute (TGEN), Phoenix, AZ 85004, USA

**Keywords:** GZ17-6.02, axitinib, renal cell carcinoma, autophagy, HDAC

## Abstract

GZ17-6.02 is undergoing clinical evaluation in solid tumors and lymphoma. The present studies were performed to define its biology in renal carcinoma cells and to determine whether it interacted with axitinib to enhance tumor cell killing. GZ17-6.02 interacted in an arithmetically greater than additive fashion with axitinib to kill kidney cancer cells. GZ17-6.02 and axitinib cooperated to inactivate ERBB2, c-MET, c-KIT, c-SRC, the AMPK, STAT3, STAT5 and eIF2α and to activate PERK, ULK1 and ATG13. The drugs interacted to increase the expression of FAS-L and to decrease the levels of MCL1, BCL-XL, and HDACs 1–3. The drugs as single agents inactivated the Hippo pathway. GZ17-6.02 and axitinib interacted to enhance autophagosome formation and autophagic flux. Knock down of Beclin1, ATG5, eIF2α, toxic BH3 domain proteins or CD95/FADD significantly reduced drug combination lethality. GZ17-6.02 and axitinib increased the expression of BAK, BIM, Beclin1 and ATG5, effects blocked by knock down of eIF2α. The drugs increased phosphorylation of ULK1 S757 and ATG13 S318 and decreased the phosphorylation of mTORC1 and mTORC2, effects blocked by knock down of AMPKα. Knock down of Beclin1 or ATG5 prevented the drug combination reducing expression of HDACs 1–3 and from enhancing the expression of MHCA. Knock down of HDACs 1–3 enhanced MHCA expression. We conclude that GZ17-6.02 and axitinib interact to kill requiring ER stress signaling, autophagy and death receptor signaling. Autophagic degradation of HDACs played a key role in enhancing MHCA expression and of a potential improved response to checkpoint inhibitory immunotherapy.

## INTRODUCTION

GZ17-6.02 has three components: curcumin, harmine and isovanillin and is presently undergoing phase I safety evaluation in cancer patients with solid tumors and lymphoma (NCT03775525) [1–6]. Over the past two years we have published data demonstrating that GZ17-6.02 kills a wide range of tumor cell types, including ER+ breast, colorectal, pancreatic, hepatic, biliary, NSCLC, cutaneous melanoma, sarcoma and actinic keratoses [1–6].

In the United States approximately 74,000 new cases of renal cell carcinoma (RCC) will be diagnosed with approximately 15,000 deaths [7]. The treatment options for RCC have been revolutionized over the past 20 years. In the 1990s RCC was treated with cytotoxic chemotherapy and radiotherapy with response rates of approximately 5%, followed in the 2000s by the advent of kinase inhibitors such as Temsirolimus, everolimus, sorafenib, pazopanib and axitinib and today with the kinase inhibitors combined with checkpoint inhibitory immunotherapy. In approximately 90% of RCCs, the von Hippel-Lindau (VHL) E3 ligase is mutated or silenced [8]. A primary target of VHL is the transcription factor hypoxia inducible factor 1 alpha (HIF1α) [8, 9]. Under normoxic conditions, VHL binds to HIF1α, ubiquitinates it, resulting in transcription factor degradation. Under hypoxic conditions such as a tumor, VHL cannot bind to HIF1α, with HIF1α activating genes whose products promote angiogenesis, e.g., VEGF and PDGFβ, as well as regulating glucose metabolism [8–11]. In addition to VHL, other proteins are also observed in RCC, including MET, FLCN, TSC1, TSC2, FH and SDH [12]. TSC1 and TSC2 regulate signaling by the AMPK and by mTOR, as energy sensing modules.

Our prior molecular findings with GZ17-6.02 demonstrated that it caused DNA damage and activated an ATM-AMPK-ULK1-ATG13 pathway, concomitant with inactivation of mTORC1 and mTORC2, resulting in enhanced autophagosome formation followed by autophagic flux. Knock down of autophagy regulatory proteins such as Beclin1 or ATG5, or knock down of ATM, AMPKα or ULK1 suppressed autophagosome formation and tumor cell killing by GZ17-6.02 [1–6]. GZ17-6.02 activated PKR-like endoplasmic reticulum kinase (PERK) and increased the phosphorylation (inactivation) of eIF2α. Knock down of eIF2α reduced autophagosome formation and the degradation of cyto-protective BH3 domain proteins such as MCL1 and BCL-XL. In addition, GZ17-6.02 increased expression of FAS-L and of toxic BH3 domain proteins, such that knock down of CD95 or the expression of the toxic BH3 domain proteins also reduced drug lethality. GZ17-6.02 interacted with 5FU to kill GI and breast cancer cells; it interacted with pemetrexed and with osimertinib to kill NSCLC cells and with dabrafenib/trametinib to kill cutaneous melanoma cells.

The present studies were designed to investigate the biology of GZ17-6.02 in VHL mutant RCC, and to define its interaction with the standard of care RCC therapeutic, the multi-kinase inhibitor axitinib.

## RESULTS

**Figure 1:**
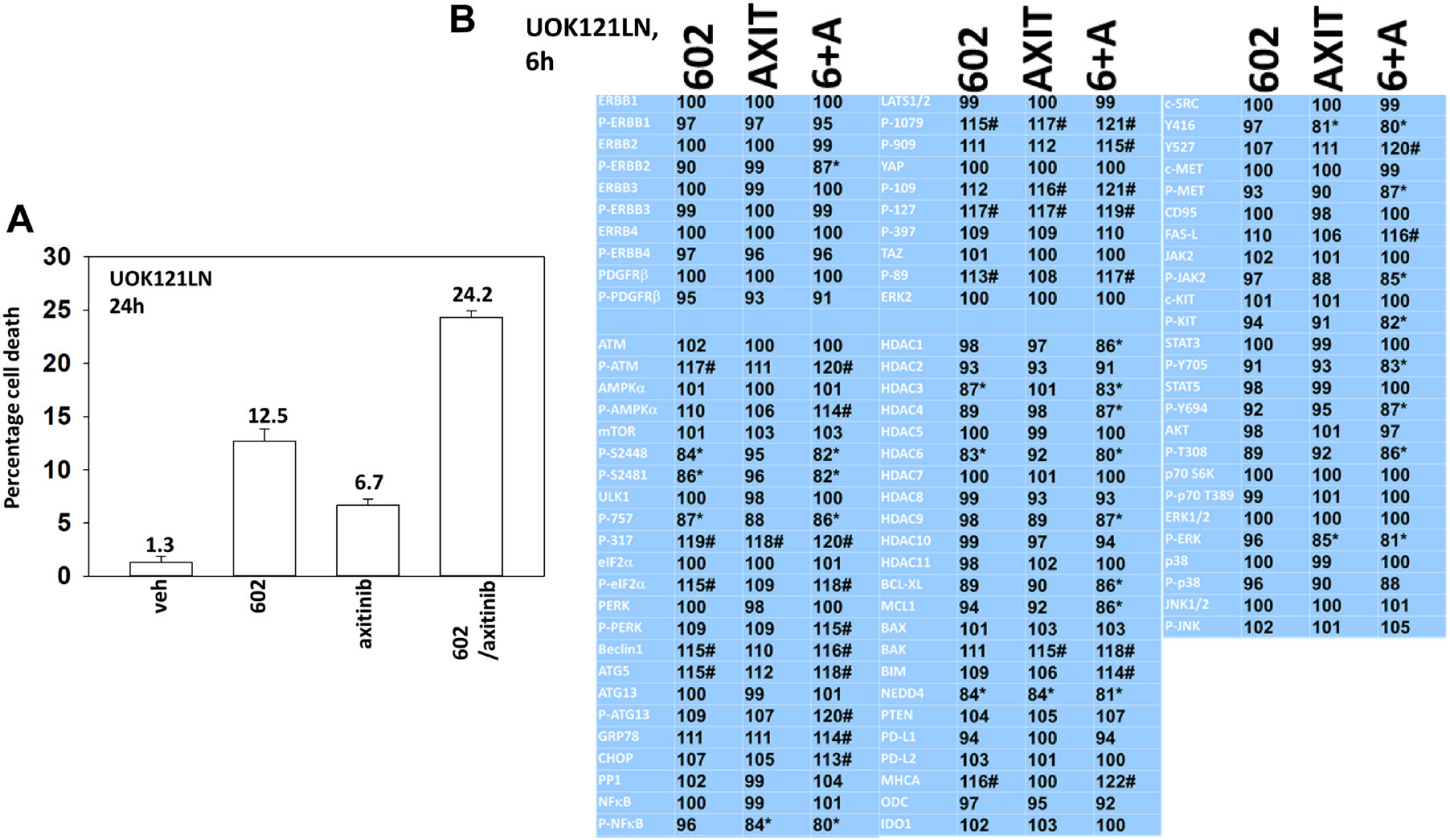
GZ17-6.02 and axitinib regulate protein expression and protein phosphorylation in UOK121LN RCCs. (**A**) UOK121LN cells were treated with vehicle control, GZ17-6.02 (2 μM), axitinib (50 nM) or the drugs in combination for 24 h. Cells were isolated, and viability determined by trypan blue exclusion. (*n* = 3 +/− SD). (**B**) UOK121LN cells were treated with vehicle control, GZ17-6.02 (2 μM), axitinib (50 nM) or the drugs in combination for 6 h. Cells were fixed in place and in-cell immunostaining performed to determine alterations in protein expression and protein phosphorylation with ERK2 as an invariant loading control (*n* = 3 +/− SD). ^*^ *p* < 0.05 less than vehicle control; ^#^ *p* < 0.05 greater than vehicle control.

**Figure 2:**
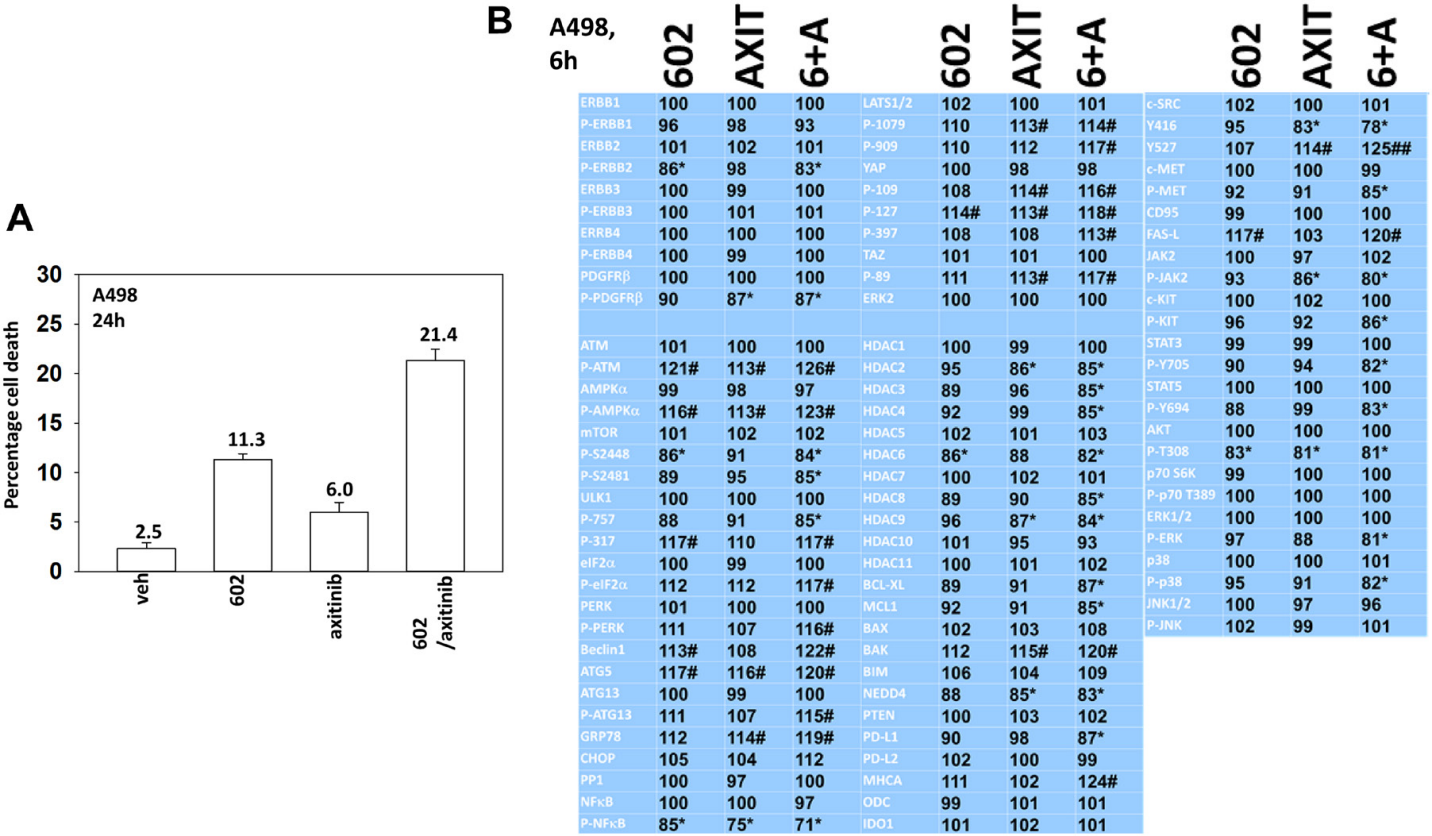
GZ17-6.02 and axitinib regulate protein expression and protein phosphorylation in A498 RCCs. (**A**) A498 cells were treated with vehicle control, GZ17-6.02 (2 μM), axitinib (50 nM) or the drugs in combination for 24 h. Cells were isolated, and viability determined by trypan blue exclusion. (*n* = 3 +/− SD). (**B**) A498 cells were treated with vehicle control, GZ17-6.02 (2 μM), axitinib (50 nM) or the drugs in combination for 6 h. Cells were fixed in place and in-cell immunostaining performed to determine alterations in protein expression and protein phosphorylation with ERK2 as an invariant loading control (*n* = 3 +/− SD). ^*^ *p* < 0.05 less than vehicle control; ^#^ *p* < 0.05 greater than vehicle control.

We performed studies to define the biology of GZ17-6.02 and axitinib. We first determined whether axitinib interacted with GZ17-6.02 to kill RCCs lacking VHL function (Figures 1A and 2A). GZ17-6.02 interacted with axitinib in an arithmetically greater than additive fashion to kill A498 and UOK121LN cells. We also examined the alterations in intracellular signaling caused by GZ17-6.02 and axitinib. GZ17-6.02 and axitinib cooperated to inactivate ERBB2, c-MET, c-KIT, c-SRC, the AMPK, STAT3, STAT5 and eIF2α and to activate PERK, ULK1 and ATG13 (Figures 1B and 2B). The drugs interacted to increase the expression of FAS-L and to decrease the levels of MCL1, BCL-XL, and HDACs 1–3. The drugs as single agents inactivated the Hippo pathway as judged by increased YAP/TAZ phosphorylation.

**Figure 3:**
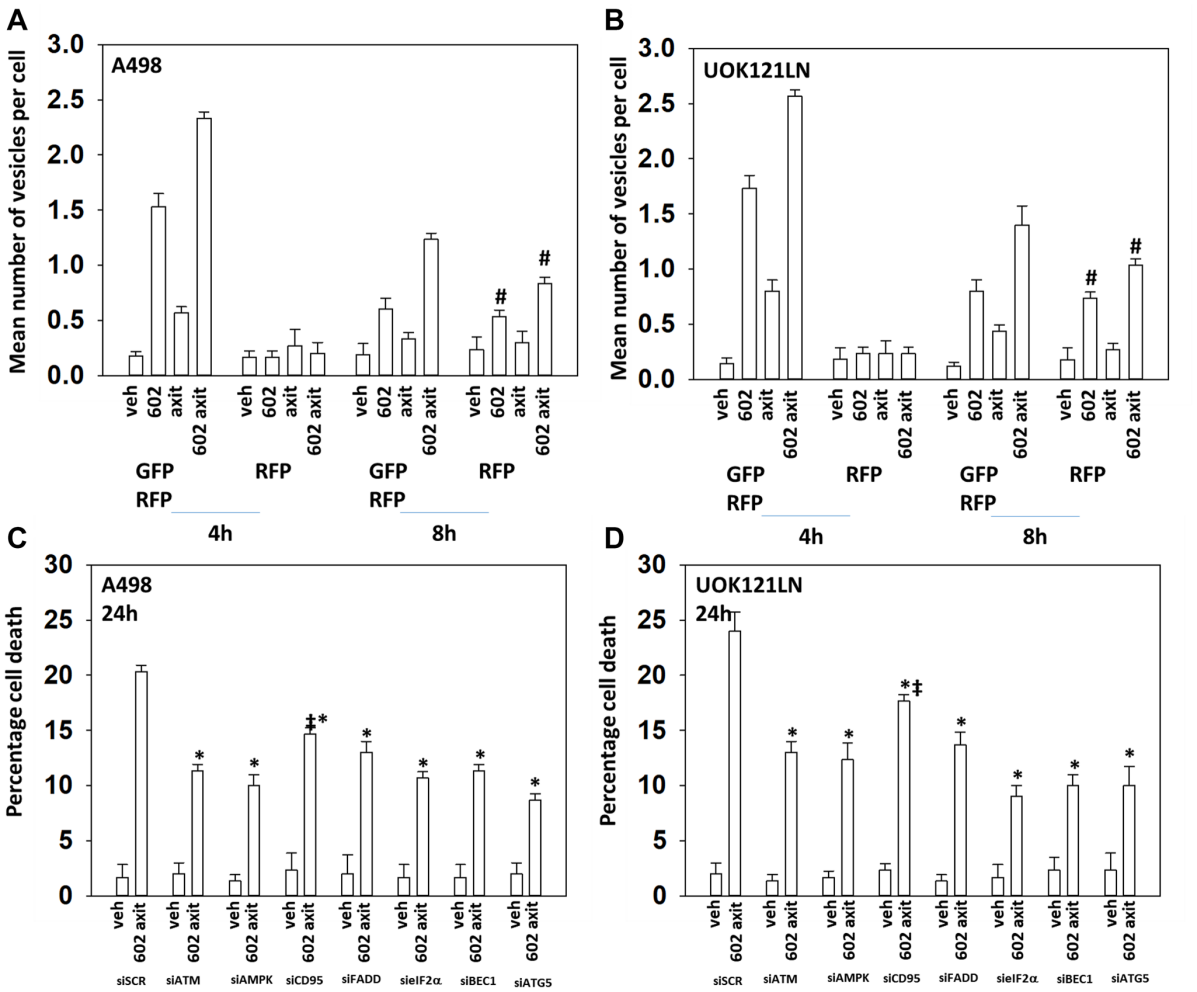
GZ17-6.02 and axitinib interact to cause toxic autophagosome formation and autophagic flux. (**A**) and (**B**) A498 and UOK121LN cells were transfected with a plasmid to express LC3-GFP-RFP. After 24 h, cells were treated with vehicle control, GZ17-6.02 (2 μM), axitinib (50 nM) or the in combination for 4 h and 8 h. The mean number of intense GFP+RFP+ and RFP+ punctae per cell was determined at each time point (*n* = 3 +/− SD) ^#^ *p* < 0.05 greater than corresponding value at the 4 h timepoint. (**C**) and (**D**) A498 and UOK121LN cells were transfected with a scrambled siRNA or with siRNA molecules to knock down expression of the indicated proteins. After 24 h, cells were treated with vehicle control or [GZ17-6.02 (2 μM) plus axitinib (50 nM)] in combination for 24 h. Cells were isolated, and viability determined by trypan blue exclusion. (*n* = 3 +/− SD). ^*^ *p* < 0.05 less than corresponding value in siSCR cells; ^‡^ *p* < 0.05 greater than corresponding siATM and siAMPK values.

**Figure 4:**
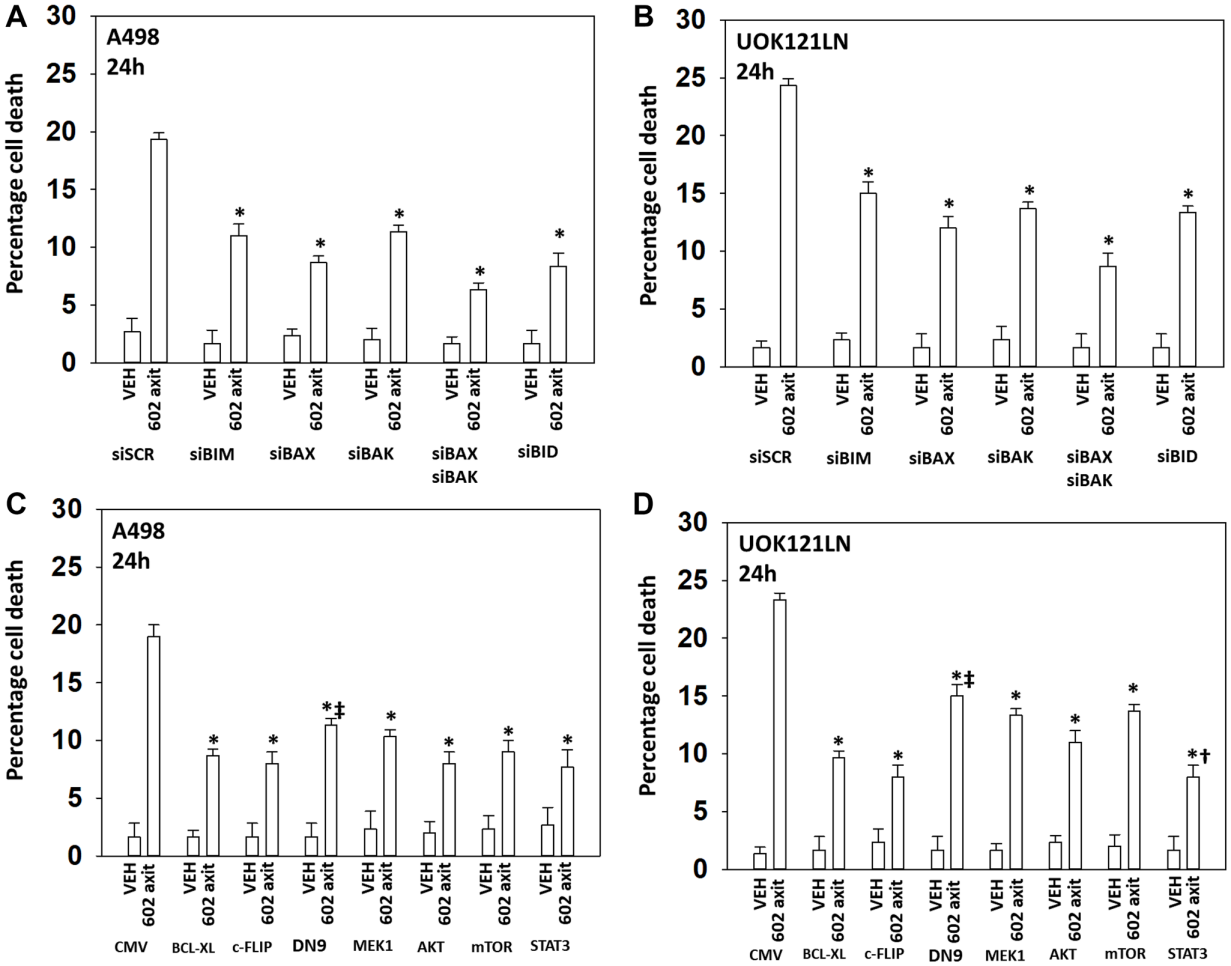
[GZ17-6.02 + axitinib] kill RCCs via death receptor signaling and mitochondrial dysfunction; the majority of killing downstream is caspase-independent. (**A**) and (**B**) A498 and UOK121LN cells were transfected with a scrambled siRNA or with siRNA molecules to knock down expression of the indicated proteins. After 24 h, cells were treated with vehicle control or [GZ17-6.02 (2 μM) plus axitinib (50 nM)] in combination for 24 h. Cells were isolated, and viability determined by trypan blue exclusion. (*n* = 3 +/− SD). ^*^ *p* < 0.05 less than corresponding value in siSCR cells. (**C**) and (**D**) A498 and UOK121LN cells were transfected with an empty vector plasmid (CMV) or with plasmids to express BCL-XL, FLIP-s, dominant negative caspase 9, activated MEK1, activated AKT, activated mTOR or activated STAT3. After 24 h, cells were treated with vehicle control or [GZ17-6.02 (2 μM) plus axitinib (50 nM)] in combination for 24 h. Cells were isolated, and viability determined by trypan blue exclusion. (*n* = 3 +/− SD). ^*^ *p* < 0.05 less than corresponding value in CMV cells; ^‡^ *p* < 0.05 greater than corresponding values in cells transfected to express BCL-XL or FLIP-s; ^†^ *p* < 0.05 less than corresponding values in cells transfected to express activated MEK1, activated AKT or activated mTOR.

Based on the data in Figures 1 and 2 we performed studies to assess macroautophagy and the mechanisms of tumor cell killing. GZ17-6.02 and axitinib interacted in an additive fashion to enhance autophagosome formation (Figure 3A and 3B). Over time, the numbers of autophagosomes declined and the numbers of autolysosomes increased, demonstrating autophagic flux. Knock down of ATM, AMPKα, CD95, FADD, eIF2α, Beclin1 or ATG5 all significantly reduced the ability of GZ17-6.02 combined with axitinib to kill RCCs (Figure 3C and 3D). CD95 knock down offered less protection than did knock down of either ATM or AMPKα. Knock down of BAX, BAK, BID or BIM each significantly reduced drug combination lethality (Figure 4A). Over-expression of BCL-XL or of FLIP-s, to a greater extent than expression of dominant negative caspase 9, significantly reduced drug combination lethality (Figure 4B). Expression of activated MEK1, activated AKT, activated mTOR or activated STAT3 all also reduced tumor cell killing by the drug combination, with expression of activated STAT3 trending towards being the most protective (Figure 4C and 4D).

**Figure 5:**
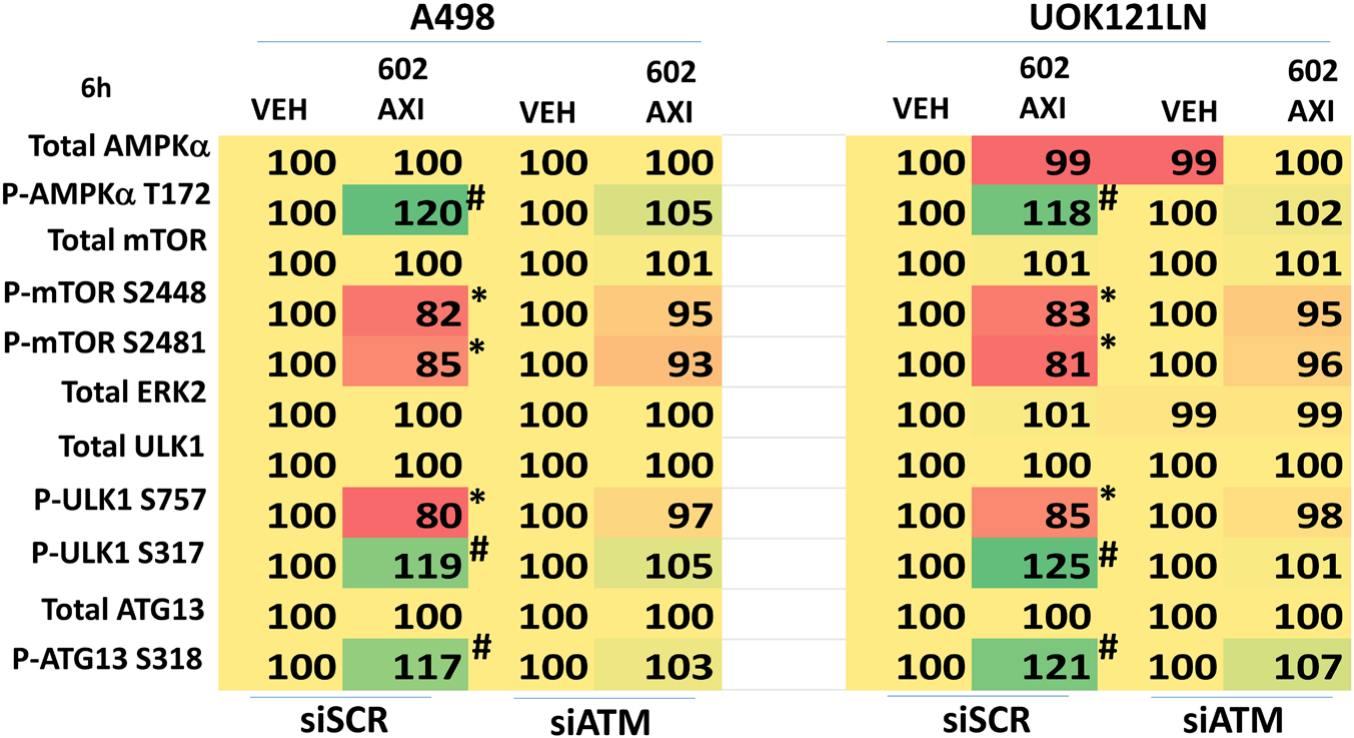
Knock down of ATM prevents [GZ17-6.02 + axitinib] regulating the phosphorylation of mTOR, ULK1 and ATG13. A498 and UOK121LN cells were transfected with a control siRNA or with an siRNA to knock down the expression of ATM. After 24 h, cells were treated with vehicle control or [GZ17-6.02 (2 μM) plus axitinib (50 nM)] in combination for 6 h. Cells were fixed in place and the expression and phosphorylation of ULK1, ATG13, mTORC1, mTORC2 and ERK2 determined (*n* = 3 +/− SD) ^*^ *p* < less than corresponding value in siSCR cells; ^#^ *p* < greater than corresponding value in siSCR cells.

**Figure 6:**
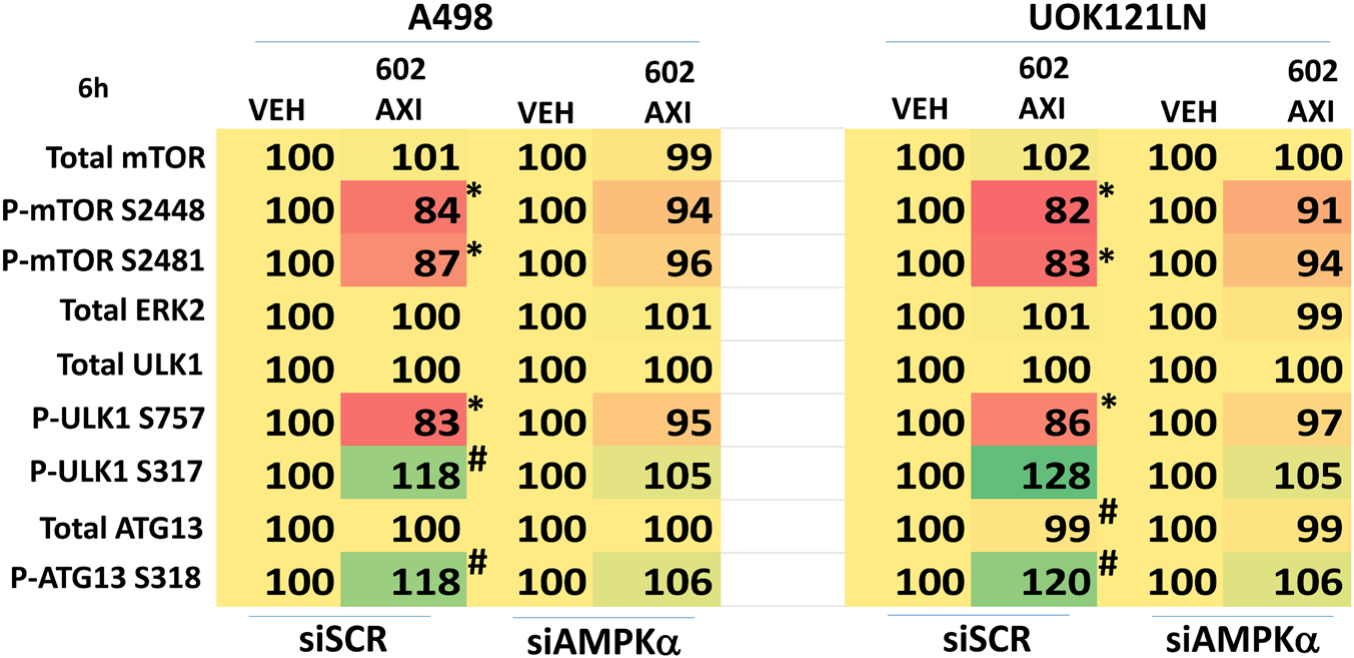
Knock down of AMPKα prevents [GZ17-6.02 + axitinib] regulating the phosphorylation of mTOR, ULK1 and ATG13. A498 and UOK121LN cells were transfected with a control siRNA or with an siRNA to knock down the expression of AMPKα. After 24 h, cells were treated with vehicle control or [GZ17-6.02 (2 μM) plus axitinib (50 nM)] in combination for 6 h. Cells were fixed in place and the expression and phosphorylation of ULK1, ATG13, mTORC1, mTORC2 and ERK2 determined (*n* = 3 +/− SD) ^*^ *p* < less than corresponding value in siSCR cells; ^#^ *p* < greater than corresponding value in siSCR cells.

**Figure 7:**
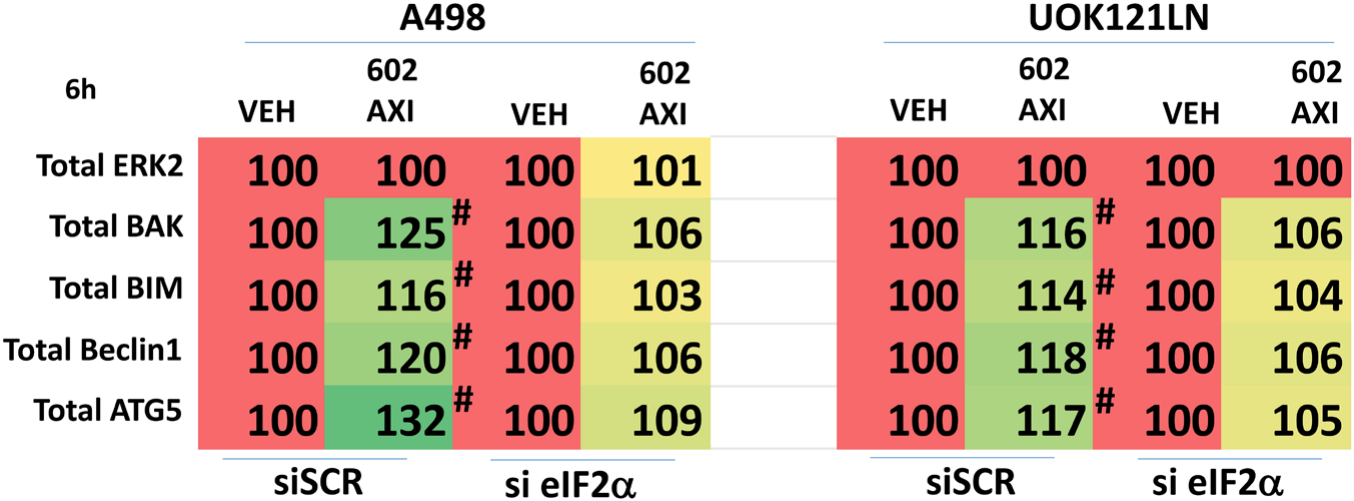
Knock down of eIF2α prevents [GZ17-6.02 + axitinib] increasing the expression of BAK, BIM, Beclin1 and ATG5. A498 and UOK121LN cells were transfected with a control siRNA or with an siRNA to knock down the expression of eIF2α. After 24 h, cells were treated with vehicle control or [GZ17-6.02 (2 μM) plus axitinib (50 nM)] in combination for 6 h. Cells were fixed in place and the expression of BAK, BIM, Beclin1, ATG5 and ERK2 determined (*n* = 3 +/− SD) ^*^ *p* < less than corresponding values in siSCR cells.

**Figure 8:**
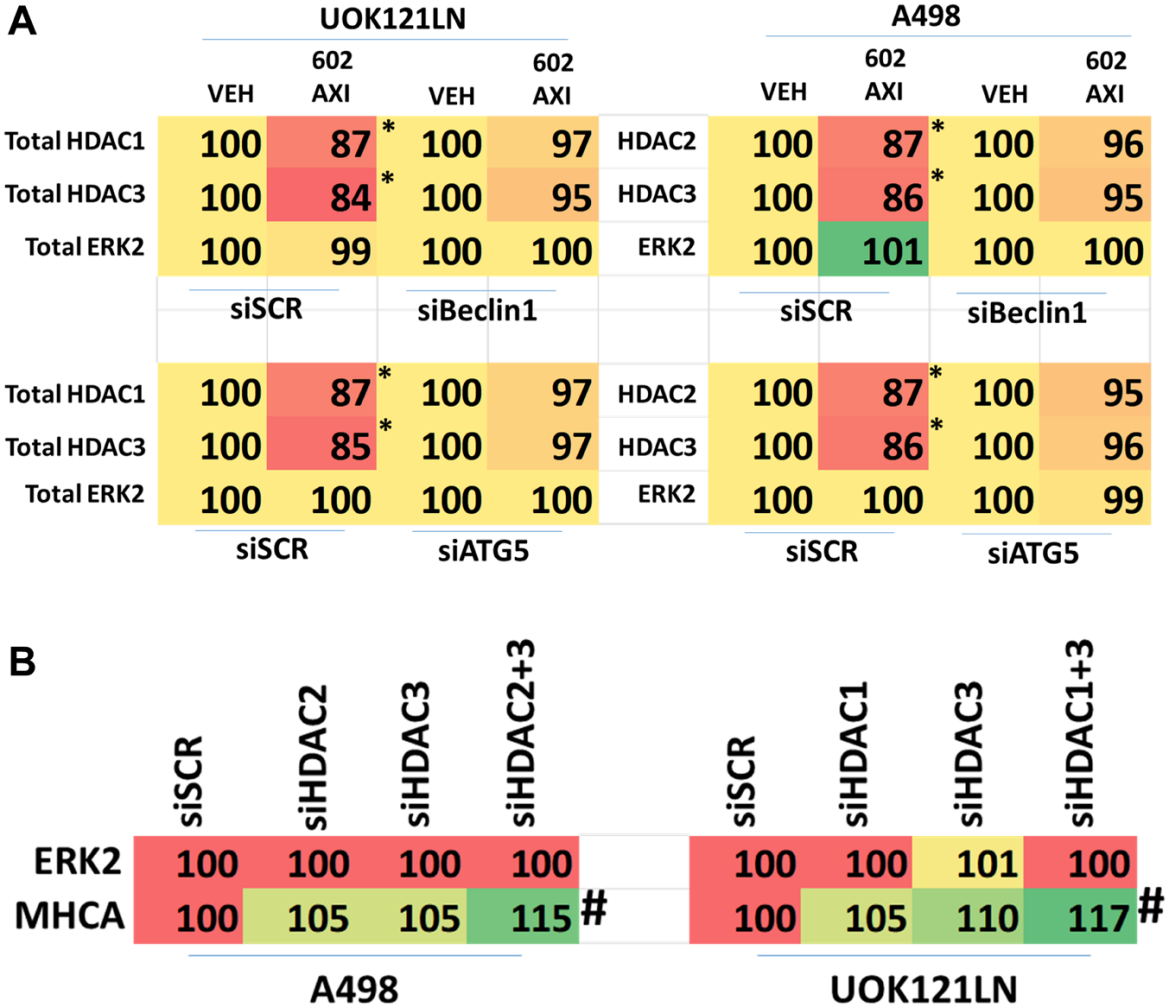
Knock down of [HDAC2 + HDAC3] or [HDAC1 + HDAC3] regulates MHCA expression in RCCs. (**A**) A498 and UOK121LN cells were transfected with a scrambled siRNA control or with siRNA molecules to knock down the expression of Beclin1 or ATG5. After 24 h, cells were treated with vehicle control or [GZ17-6.02 (2 μM) plus axitinib (50 nM)] in combination for 6 h. Cells were fixed in place and the expression of HDAC1, HDAC2, HDAC3, MHCA and ERK2 determined (*n* = 3 +/− SD) ^*^ *p* < less than corresponding values in siSCR cells. (**B**) A498 and UOK121LN cells were transfected with a scrambled siRNA control or with siRNA molecules to knock down the expression of HDAC1, HDAC2 and HDAC3, as indicated. After 24 h, cells were fixed in place and the expression of MHCA and ERK2 determined (*n* = 3 +/− SD) ^#^ *p* < 0.05 greater than siSCR control.

**Figure 9:**
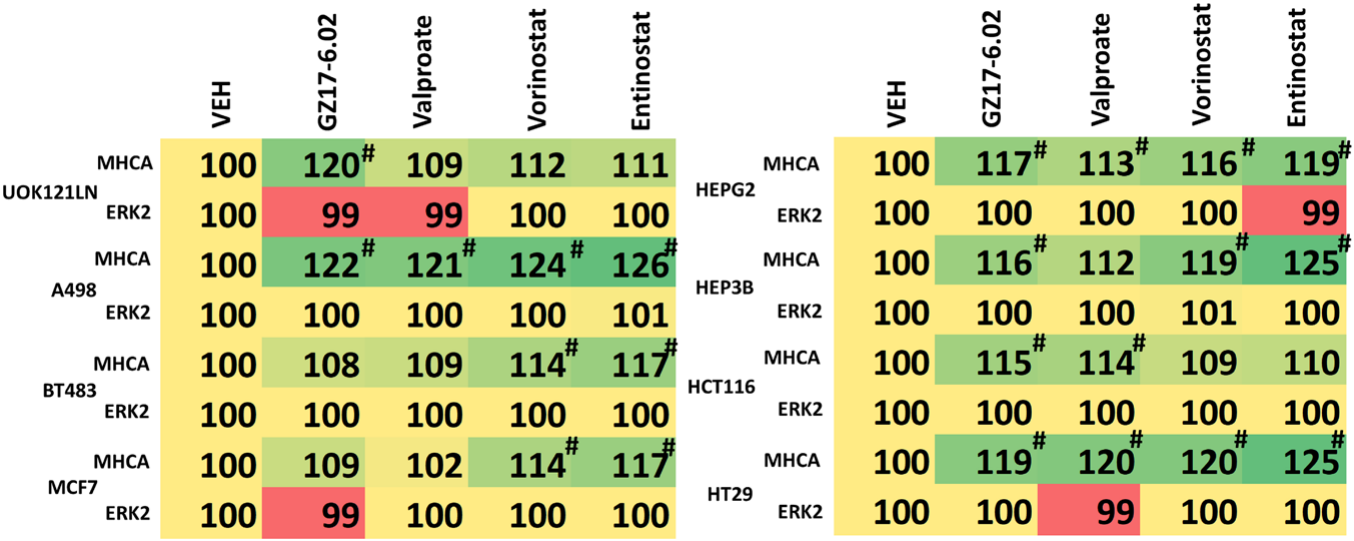
GZ17-6.02 regulates MHCA expression in a variety of tumor cell types and is more capable of regulating MHCA levels than HDAC inhibitors. Tumor cells (renal: UOK121LN, A498; breast: BT483, MCF7; hepatoma: HEPG2, HEP3B; colorectal: HCT116, HT29) were treated with vehicle control or with GZ17-6.02 (2 μM), sodium valproate (250 μM), vorinostat (250 nM) or entinostat (50 nM) for 6 h. The expression of MHCA and ERK2 determined (*n* = 3 +/− SD) ^#^ *p* < 0.05 greater than siSCR control.

Knock down of ATM or the AMPKα prevented [GZ17-6.02 + axitinib] from inactivating mTORC1 and mTORC2 and from activating ULK1, including downstream phosphorylation of ATG13 (Figures 5 and 6). Knock down of eIF2α prevented GZ17-6.02 and axitinib from increasing the expression of BAK, BIM, Beclin1 and ATG5 (Figure 7). In Figures 1 and 2 we noted that in A498 cells the drug combination reduced the expression of HDAC2 and HDAC3 whereas in UOK121LN cells, it lowered the levels of HDAC1 and HDAC2. Knock down of Beclin1 or ATG5 prevented the drug combination reducing expression of HDACs 1, 2 and 3 and from enhancing the expression of the Class I HLA, MHCA (Figure 8A and 8B). In A498 cells, knock down of HDAC2 and HDAC3 increased MHCA expression whereas in UOK121LN cells, knock down of HDAC1 and HDAC3 enhanced MHCA expression (Figure 8B). Finally, based on the data in the earlier Figures, we determined whether HDAC inhibitors could alter the expression of MHCA in RCC cells and other tumor cell types. In A498 cells, GZ17-6.02 increased the expression of MHCA as did the HDAC inhibitors sodium valproate, vorinostat and entinostat (Figure 9). However, in UOK121LN cells only GZ17-6.02 enhanced MHCA levels, and not the HDAC inhibitors. In BT483 and MCF7 breast cancer cells, vorinostat and entinostat both increased MHCA expression. In hepatoma cells, GZ17-6.02 and HDAC inhibitors all could elevate MHCA expression with similar data also found in colon cancer cells. We conclude that GZ17-6.02 and axitinib interact to kill requiring ER stress signaling, autophagy and death receptor signaling. Autophagic degradation of HDACs played a key role in enhancing MHCA expression and of a potential improved response to checkpoint inhibitory immunotherapy.

## DISCUSSION

The present studies demonstrated that GZ17-6.02 interacted with the multi-kinase inhibitor axitinib to kill renal carcinoma cells. GZ17-6.02 and axitinib increased the phosphorylation of AMPKα T172 and ATG13 S318 and reduced that of mTOR in an ATM-dependent fashion. Knock down of AMPKα prevented increased ATG13 S318 phosphorylation and the inactivation of mTOR. The drug combination activated PERK and inactivated eIF2α; the observed increases in Beclin1 and ATG5 expression after GZ17-6.02 and axitinib treatment were prevented by knock down of eIF2α. Knock down of ATM, AMPKα, ATG5, Beclin1 or eIF2α significantly reduced the lethality of [GZ17-6.02 + axitinib]. Death receptor signaling from CD95/FADD and the actions of BAX, BAK, BIM and BID also contributed to the killing process. Signaling by ATM-AMPK was more important in the causation of cell killing than that of CD95. Knock down of eIF2α prevented the drug combination from increasing the expression of BAK and BIM. Compared to over-expression of FLIP-s or BCL-XL, expression of dominant negative caspase 9 was less effective at preventing tumor cell killing. Thus, the molecular mechanisms by which GZ17-6.02 interacted with axitinib to cause tumor cell death were similar to data combining GZ17-6.02 with other cancer therapeutics such as 5FU, including death receptor signaling, autophagosome formation and autophagic flux, and mitochondrial dysfunction with both apoptotic and non-apoptotic downstream killing.

These present studies have several limitations that should be noted. We have not performed any studies in non-transformed kidney cells and the tumor cells were cultured in 2D. At present, due to restrictions on performing animal studies because of the SARS-CoV-2 pandemic, we have been unable to perform *in vivo* analyses in tumors, conforming our *in vitro* findings.

In both A498 and UOK121LN cells, [GZ17-6.02 + axitinib] increased MHCA expression, which *in vivo* would be predictive for an enhanced response to immunotherapy. In UOK121LN cells the drug combination reduced expression of HDAC1, HDAC3 and HDAC6. In A498 cells the drug combination lowered the levels of HDAC2, HDAC3 and HDAC6. Knock down of ATG5 or Beclin1 prevented the degradation of HDACs1/2/3. Knock down of [HDAC1 + HDAC3] in UOK121LN cells and [HDAC2 + HDAC3] in A498 cells increased the expression of MHCA. Thus, the ATM-AMPK signaling module, which regulates autophagosome formation, also indirectly controls the expression of MHCA, potentially enhancing the effects of checkpoint inhibitory immunotherapy.

We compared the actions of GZ17-6.02 on MHCA expression to those of several HDAC inhibitors in a variety of different tumor cell types. Other than in breast cancer cells, GZ17-6.02 as a single agent significantly increased MHCA expression. In contrast, the ability of HDAC inhibitors to increase MHCA levels was variable, with some, but not all HDAC inhibitors capable of elevating MHCA expression and with variability even within a particular tumor cell type. The utility of HDAC inhibitors as cancer therapeutics has unfortunately not lived up to early expectations based on pre-clinical data, and the negative sequelae of this class of drug has also hindered their use in the clinic. Initial findings from the phase I trial of GZ17-6.02 (NCT03775525) indicate that it is well-tolerated by patients with modest Grade 1 toxicities. Based on our prior *in vivo* data with GZ17-6.02 and checkpoint inhibitory immunotherapy in CT26 mouse colon cancer cells, this suggests GZ17-6.02 may have utility in enhancing the immune response of RCC tumors treated with nivolumab plus ipilimumab, axitinib plus pembrolizumab, or avelumab plus pembrolizumab.

## MATERIALS AND METHODS

### Materials

**Figure 10:**
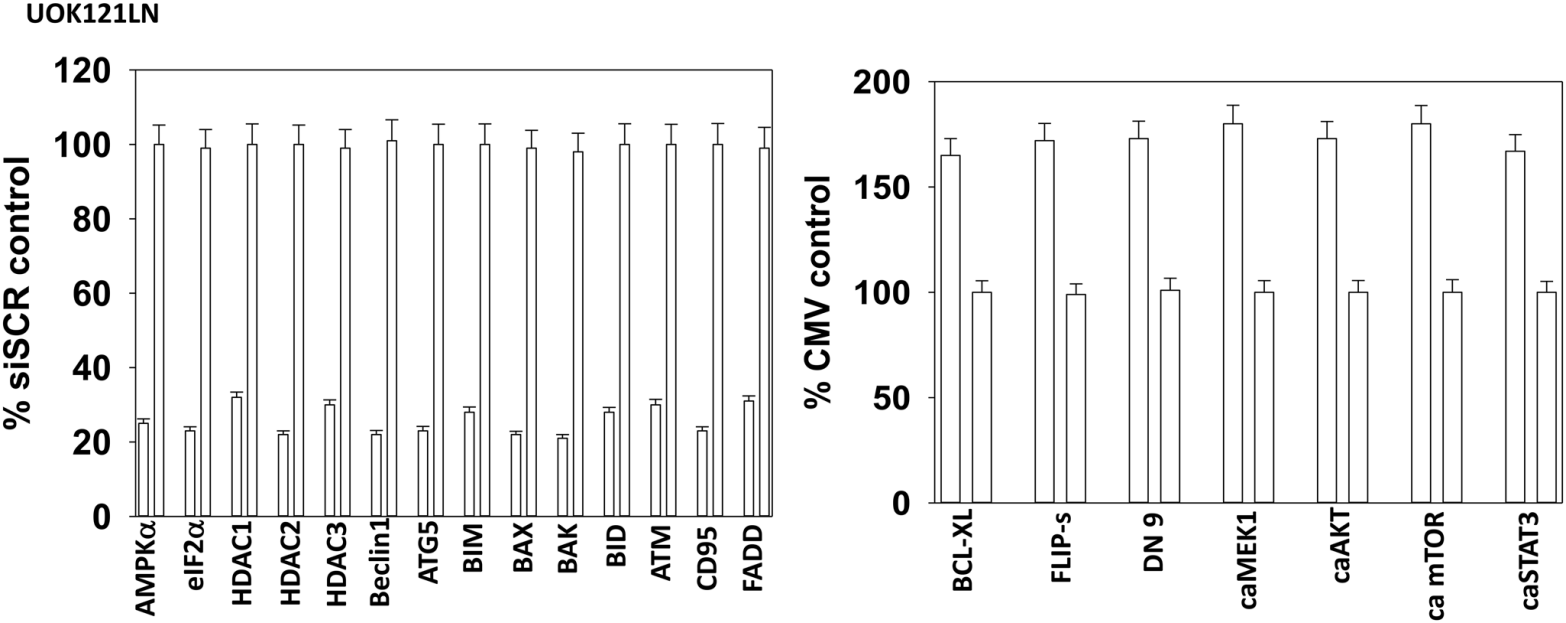
Control siRNA knock down and protein over-expression in RCCs. Cells were transfected to over-express or knock down protein expression. Twenty-four h after transfection, cells were fixed in place and immuno-stained to determine protein levels with invariant ERK2 as a loading control (*n* = 3 +/− SD).

The A498 cell line was obtained from the ATCC (Bethesda, MD). The UOK121LN cell isolate was kindly supplied by Dr. Marston Linehan (Nation Cancer Institute, Bethesda, MD) Axitinib was purchased from Selleckchem (Houston, TX). All Materials were obtained as described in the references [1–6]. Trypsin-EDTA, DMEM, RPMI, penicillin-streptomycin were purchased from GIBCOBRL (GIBCOBRL Life Technologies, Grand Island, NY). Other reagents and performance of experimental procedures were as described [1–6]. Antibodies were purchased from Cell Signaling Technology (Danvers, MA); Abgent (San Diego, CA); Novus Biologicals (Centennial, CO); Abcam (Cambridge, UK); and Santa Cruz Biotechnology (Dallas, TX). Specific multiple independent siRNAs to knock down the expression of CD95, FADD, Beclin1, ATG5 AMPKα_1_, ATM, BIM, BAX, BAK, BID and eIF2α, and scramble control, were purchased from Qiagen (Hilden, Germany) and Thermo Fisher (Waltham, MA). Control studies were presented showing on-target specificity of our siRNAs, primary antibodies, and our phospho-specific antibodies to detect both total protein levels and phosphorylated levels of proteins [1–6] (Figure 10).

### Methods

All bench-side Methods used in this manuscript have been performed and described in the peer-reviewed references [1–6].

### Assessments of protein expression and protein phosphorylation [1–6]

At various time-points after the initiation of drug exposure, cells are fixed in place using paraformaldehyde and using Triton X100 for permeabilization. Standard immunofluorescent blocking procedures are employed, followed by incubation of different wells with a variety of validated primary antibodies and subsequently validated fluorescent-tagged secondary antibodies are added to each well. The microscope determines the background fluorescence in the well and in parallel randomly determines the mean fluorescent intensity of 100 cells per well.

### Detection of cell death by trypan blue assay [1–6]

Cells were treated with vehicle control or with drugs alone or in combination for 24 h. At the indicated time points cells were harvested by trypsinization and centrifugation. Cell pellets were resuspended in PBS and mixed with trypan blue agent. Viability was determined microscopically using a hemocytometer. Five hundred cells from randomly chosen fields were counted and the number of dead cells was counted and expressed as a percentage of the total number of cells counted.

### Transfection of cells with siRNA or with plasmids [1–6]

Cells were plated and 24 h after plating, transfected. Plasmids to express FLIP-s, BCL-XL, dominant negative caspase 9, activated AKT, activated STAT3, activated mTOR and activated MEK1 EE were used throughout the study (Addgene, Waltham, MA, USA). Empty vector plasmid (CMV) was used as a control. For siRNA transfection, 10 nM of the annealed siRNA or the negative control (a “scrambled” sequence with no significant homology to any known gene sequences from mouse, rat or human cell lines) were used.

### Assessments of autophagosome and autolysosome levels [1–6]

Cells were transfected with a plasmid to express LC3-GFP-RFP (Addgene, Watertown, MA, USA). Twenty-four hs after transfection, cells are treated with vehicle control or the drugs alone or in combination. Cells were imaged and recorded at 60X magnification 4 hs and 8 hs after drug exposure and the mean number of GFP+ and RFP+ punctae per cell determined from >50 randomly selected cells per condition.

### Data analysis

Comparison of the effects of various treatments was using one-way ANOVA for normalcy followed by a two tailed Student’s *t*-test with multiple comparisons. Differences with a *p*-value of < 0.05 were considered statistically significant. Experiments are the means of multiple individual data points per experiment from 3 independent experiments (± SD).

## Abbreviations

ERK: extracellular regulated kinase
PI3K: phosphatidyl inositol 3 kinase
ca: constitutively active
dn: dominant negative
ER: endoplasmic reticulum
AMPK: AMP-dependent protein kinase
mTOR: mammalian target of rapamycin
JAK: Janus Kinase
STAT: Signal Transducers and Activators of Transcription
MAPK: mitogen activated protein kinase
PTEN: phosphatase and tensin homologue on chromosome ten
ROS: reactive oxygen species
CMV: empty vector plasmid
si: small interfering
SCR: scrambled
VEH: vehicle
602: GZ17-6.02
axitinib: axit
PD-L1: programed death ligand 1
MHCA: major histocompatibility class A
HDAC: histone deacetylase

## References

[1] Booth L , Roberts JL , West C , Von Hoff D , Dent P . GZ17-6.02 initiates DNA damage causing autophagosome-dependent HDAC degradation resulting in enhanced anti-PD1 checkpoint inhibitory antibody efficacy. J Cell Physiol. 2020; 235:8098–113. 10.1002/jcp.29464. 31951027

[2] Booth L , West C , Hoff DV , Dent P . GZ17-6.02 and Doxorubicin Interact to Kill Sarcoma Cells via Autophagy and Death Receptor Signaling. Front Oncol. 2020; 10:1331. 10.3389/fonc.2020.01331. 32983965 PMC7492267

[3] Booth L , West C , Von Hoff D , Kirkwood JM , Dent P . GZ17-6.02 Interacts With [MEK1/2 and B-RAF Inhibitors] to Kill Melanoma Cells. Front Oncol. 2021; 11:656453. 10.3389/fonc.2021.656453. 33898322 PMC8061416

[4] West CE , Kwatra SG , Choi J , Von Hoff D , Booth L , Dent P . A novel plant-derived compound is synergistic with 5-fluorouracil and has increased apoptotic activity through autophagy in the treatment of actinic keratoses. J Dermatolog Treat. 2020 May 20. 10.1080/09546634.2020.1764905. . [Epub ahead of print]. 32362152

[5] Booth L , West C , Moore RP , Von Hoff D , Dent P . GZ17-6.02 and Pemetrexed Interact to Kill Osimertinib-Resistant NSCLC Cells That Express Mutant ERBB1 Proteins. Front Oncol. 2021; 11:711043. 10.3389/fonc.2021.711043. 34490108 PMC8417372

[6] Booth L , West C , Moore RP , Von Hoff D , Dent P . GZ17-6.02 and palbociclib interact to kill ER+ breast cancer cells. Oncotarget. 2022; 13:92–104. 10.18632/oncotarget.28177. 35035775 PMC8754587

[7] Key Statistics About Kidney Cancer. American Cancer Society. https://www.cancer.org/cancer/kidney-cancer/about/key-statistics.html.

[8] Gulati S , Vogelzang NJ . Biomarkers in renal cell carcinoma: Are we there yet? Asian J Urol. 2021; 8:362–75. 10.1016/j.ajur.2021.05.013. 34765444 PMC8566366

[9] Sato Y , Yoshizato T , Shiraishi Y , Maekawa S , Okuno Y , Kamura T , Shimamura T , Sato-Otsubo A , Nagae G , Suzuki H , Nagata Y , Yoshida K , Kon A , et al. Integrated molecular analysis of clear-cell renal cell carcinoma. Nat Genet. 2013; 45:860–67. 10.1038/ng.2699. 23797736

[10] Kaelin WG Jr . Von Hippel-Lindau disease. Annu Rev Pathol. 2007; 2:145–73. 10.1146/annurev.pathol.2.010506.092049. 18039096

[11] Bader HL , Hsu T . Systemic VHL gene functions and the VHL disease. FEBS Lett. 2012; 586:1562–69. 10.1016/j.febslet.2012.04.032. 22673568 PMC3372859

[12] Linehan WM , Srinivasan R , Schmidt LS . The genetic basis of kidney cancer: a metabolic disease. Nat Rev Urol. 2010; 7:277–85. 10.1038/nrurol.2010.47. 20448661 PMC2929006

